# Supplementary material for: Facile One-Pot Green Synthesis of Magneto-Luminescent Bimetallic Nanocomposites with Potential as Dual Imaging Agent
Source: Nanomaterials (Basel). 2023 Mar 13;13(6):1027. doi: 10.3390/nano13061027 (PMC10054767; doi:10.3390/nano13061027)
Supplement: Supplementary file 1 [file nanomaterials-13-01027-s001.zip › nanomaterials-2259043-supplementary.pdf]

Supplementary Materials

Facile One-Pot Green Synthesis of Magneto-Luminescent Bimetallic Nanocomposites with Potential as Dual Imaging Agent

Radek Ostruszka [1], Denisa Půlpánová [2], Tomáš Pluháček [3], Ondřej Tomanec [4], Petr Novák [1], Daniel Jirák [2,5] and Karolína Šišková [1,*]

[1] Department of Experimental Physics, Faculty of Science, Palacký University Olomouc, 77900 Olomouc, Czech Republic
[2] Faculty of Health Studies, Technical University of Liberec, 46117 Liberec, Czech Republic
[3] Department of Analytical Chemistry, Faculty of Science, Palacký University Olomouc, 77900 Olomouc, Czech Republic
[4] Regional Centre of Advanced Technologies and Materials, Czech Advanced Technology and Research Institute, Palacký University Olomouc, 77900 Olomouc, Czech Republic
[5] Radiodiagnostic and Interventional Radiology Department, Institute for Clinical and Experimental Medicine, 14021 Prague, Czech Republic
* Correspondence: karolina.siskova@upol.cz

Keywords: Nanocomposite materials; Gold nanoclusters; Luminescence materials; MRI assessment; SPION; bovine serum albumin.

Table of contents:

1. **3D-fluorescence maps and average quantum yield determination of AuBSA and AuBSA-Fe** (pages 2-3)
2. **Particle size distribution determined by DLS** (pages 4-6)
3. **ICP-MS method validation, determination of Au and Fe concentrations** (pages 7-8)
4. **MRI for AuBSA-Fe samples** (page 9-11)
5. **Ageing and recommended storage conditions of AuBSA-Fe samples as verified by XPS measurements** (pages 12-17)
6. **Cell viability tests** (page 18)

1. 3D-fluorescence maps and average quantum yield determination of AuBSA and AuBSA-Fe

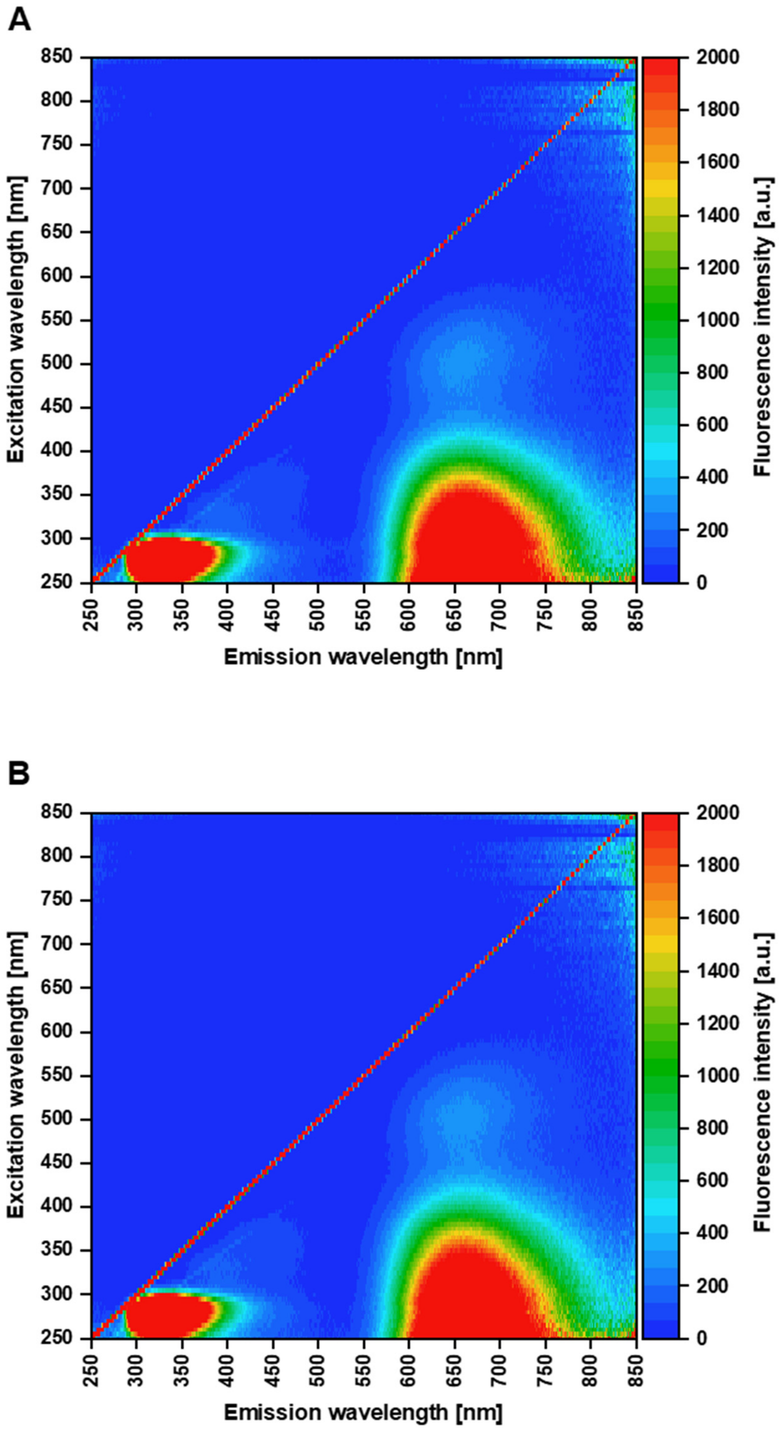

Figure S1. 3D excitation-emission maps of AuBSA (A) and AuBSA-Fe (B).

Table S1. Quantum yield and position of emission maxima of AuBSA (7 independent sample preparations).

AuBSA	I	II	III	IV	V	VI	VII	Average	SD
**Absorbance**	0.0196	0.0211	0.0204	0.0207	0.0205	0.0205	0.0206	0.0205	0.0005
**Quantum yield [%]**	6.4	6.1	6.4	6.4	6.4	6.5	6.4	6.4	0.1
**Maximum at [nm]**	659	654	656	656	658	659	656	657	2

Table S2. Quantum yield and position of emission maxima of AuBSA-Fe (7 independent sample preparations).

AuBSA-Fe	I	II	III	IV	V	VI	VII	Average	SD
**Absorbance**	0.0197	0.0202	0.0205	0.0200	0.0201	0.0207	0.0204	0.0202	0.0003
**Quantum yield [%]**	6.5	6.1	6.1	6.3	6.2	6.0	6.2	6.2	0.2
**Maximum at [nm]**	658	655	656	655	656	655	658	656	1

2. Particle size distribution determined by DLS

Particle size distribution (PSD) within a liquid sample can be determined by dynamic light scattering (DLS), i.e., by measuring changes of the scattered light intensity as a function of time. The instrument (Zetasizer Malvern) enables to determine PSD based on intensity, number, and volume. The former is the only value, which is measured experimentally; the two others are calculated from the former under certain assumptions (spherical, isolated, identical particles). Average PSD histograms of AuBSA and AuBSA-Fe nanocomposites are shown in Figure SI-2 for the sake of a direct comparison and for explanation of polydispersity (PDI) increase in AuBSA-Fe in comparison to AuBSA as shown and discussed in the main text. However, keep in mind that contents of big particles in PSD based on intensity changes of scattered light are overestimated because the bigger particles, the higher contribution of their scattering.

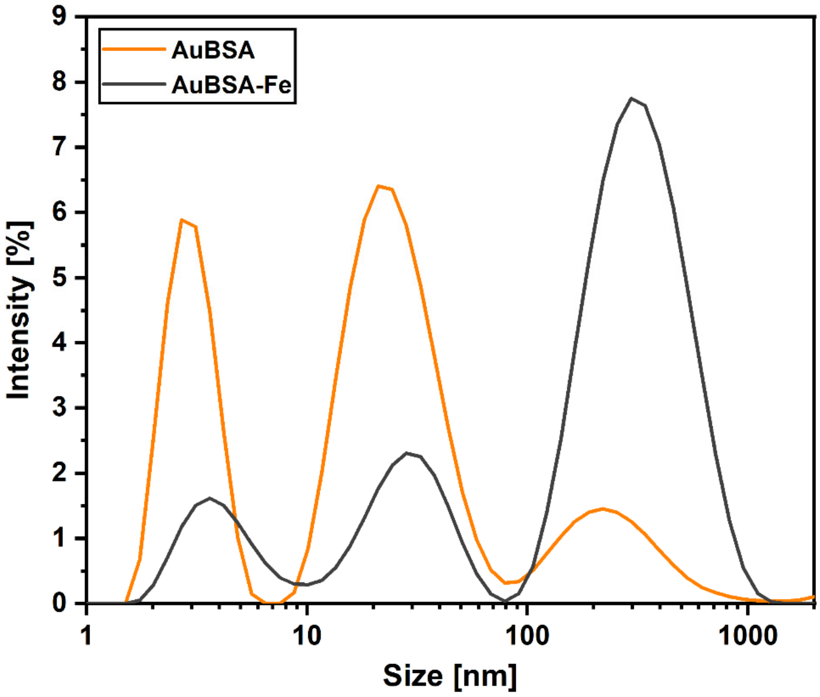

Figure S2. Particle size distribution (PSD) histograms of AuBSA (orange curve) and AuBSA-Fe (black curve) based on the changes in intensity of scattered light (633 nm laser line) measured by dynamic light scattering. Trimodal PSD is observed in both samples, however, with different average values and percentage (in brackets): 266.1 ± 38.0 nm (12.9 ± 2.1 %), 26.8 ± 2.4 nm (50.8 ± 1.2 %), 3.0 ± 0.1 nm (27.7 ± 0.8 %) for AuBSA; 351.2 ± 21. 0 nm (68.7 ± 1.4 %), 30.0 ± 2.9 nm (16.7 ± 0.9 %), 4.4 ± 0.3 nm (10.6 ± 0.8 %) for AuBSA-Fe.

Three different types of NP sizes are thus present in both aqueous systems AuBSA and AuBSA-Fe (representing proper solutions, i.e., without any aggregates visible by naked eyes): several units, tens, and hundreds of nanometers, which well explains the relatively large PDI values. Obviously, there is a more significant contribution of the largest particles (around 351 nm in average) in PSD of AuBSA-Fe in comparison to PSD of AuBSA (Figure SI-2). However, their sizes

are still in hundreds of nanometers, which means that these nanoparticles could be internalized by cells (which are 10-100 μm in size for most animal and plant cells).

Reproducibility of PSD data is demonstrated in Figures SI-3 and SI-4 and in Tables SI-3 and SI-4. By assuming spherical isolated particles, PSD based on number can be also calculated as seen in Tables SI-3 and SI-4. However, it should be reminded that PSD based on particle number is the calculated value obtained under the above-mentioned assumptions of identical, spherical, isolated particles.

Table S3. PSD of several independently measured AuBSA samples determined by DLS based on intensity (Int) and number (Num). Average and standard deviation (SD) values are then calculated.

AuBSA	Int 1 [nm]	Int 2 [nm]	Int 3 [nm]	Num 1 [nm]	Area Int 1 [%]	Area Int 2 [%]	Area Int 3 [%]	Area Num 1 [%]	Z-Average [nm]	PDI
I	243.6	26.4	3.0	2.2	13.1	52.6	27.9	100.0	42.86	0.236
II	246.2	25.0	2.9	2.2	12.0	50.7	27.7	100.0	16.08	0.438
III	303.3	27.1	3.0	2.3	13.4	49.5	27.8	100.0	21.15	0.389
IV	227.2	24.9	3.0	2.2	10.1	49.9	28.7	100.0	20.62	0.327
V	310.4	30.8	3.2	2.4	15.9	51.3	26.5	100.0	18.92	0.494
Average	266.1	26.8	3.0	2.3	12.9	50.8	27.7	100.0	23.9	0.4
SD	38.0	2.4	0.1	0.1	2.1	1.2	0.8	0.0	10.8	0.1

Table S4. PSD of several independently measured AuBSA-Fe determined by DLS based on intensity (Int) and number (Num). Average and standard deviation (SD) values are then calculated.

AuBSA-Fe	Int 1 [nm]	Int 2 [nm]	Int 3 [nm]	Num 1 [nm]	Area Int 1 [%]	Area Int 2 [%]	Area Int 3 [%]	Area Num 1 [%]	Z-Average [nm]	PDI
I	361.2	34.4	4.7	2.2	67.7	17.2	11.2	100.0	83.61	1.000
II	331.5	26.8	4.2	2.8	70.3	15.4	9.8	100.0	71.59	1.000
III	382.3	30.4	3.9	2.6	68.8	17.8	9.8	100.0	71.28	1.000
IV	334.1	28.5	4.6	2.5	67.0	16.4	11.3	100.0	68.13	1.000
V	347.0	30.0	4.5	2.4	69.8	16.9	11.1	100.0	61.61	1.000
Average	351.2	30.0	4.4	2.5	68.7	16.7	10.6	100.0	71.2	1.0
SD	21.0	2.9	0.3	0.2	1.4	0.9	0.8	0.0	8.0	0.0

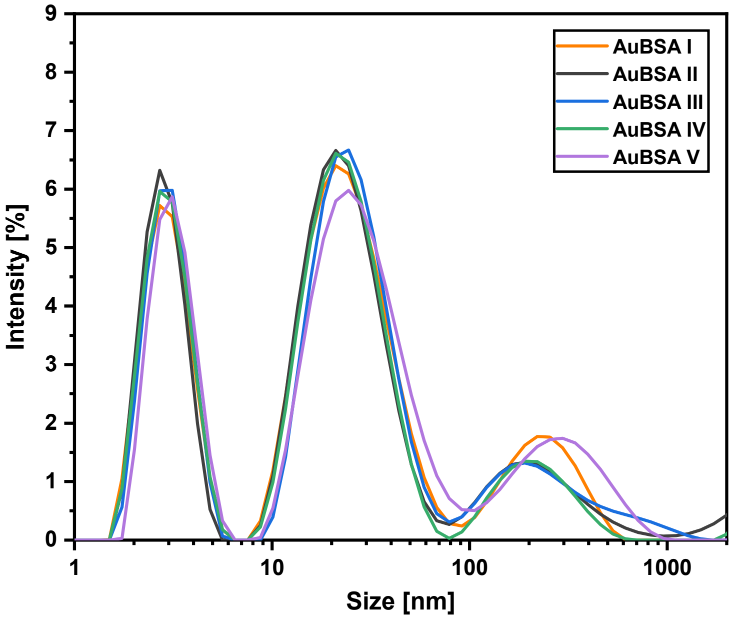

Figure S3. Histograms of PSD of several independently measured AuBSA.

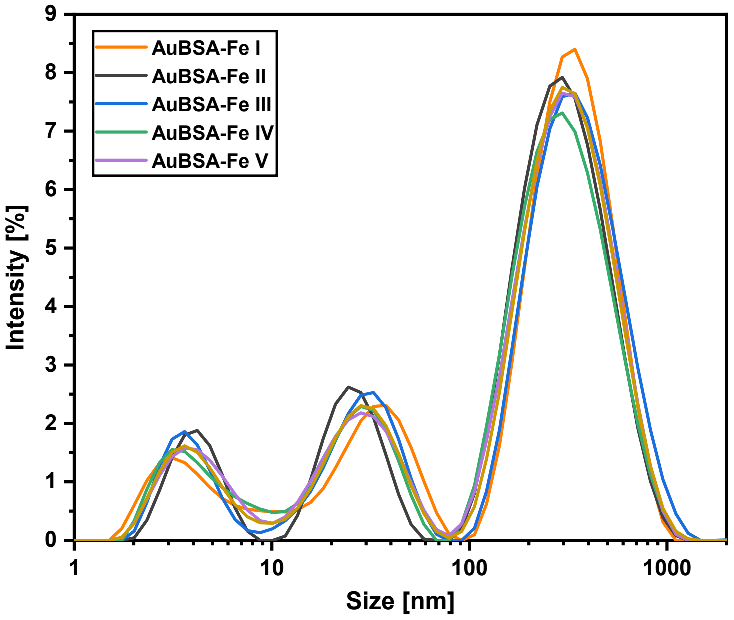

Figure S4. Histograms of PSD of several independently measured AuBSA-Fe.

3. ICP-MS method validation and determination of Au and Fe concentrations

The total gold and iron concentrations were determined by an Agilent 7700x ICP-MS (Agilent Technologies Ltd., Japan) fitted with ASX-520 autosampler, MicroMist concentric nebulizer, a Scott-type double pass spray chamber, and an octopole reaction system working in helium mode was used for all analyses. The optimized ICP-MS operating conditions were as follows: RF power of 1550 W, plasma gas flow rate of 15.0 $L \cdot min^{-1}$, an auxiliary gas flow rate of 0.9 $L \cdot min^{-1}$, nebulizer gas flow rate of 1.05 $L \cdot min^{-1}$, collision gas He flow rate of 4.3 $mL \cdot min^{-1}$ and a dwell time of 100 ms for ^{56}Fe, ^{197}Au, ^{45}Sc, ^{209}Bi isotopes (last two served as internal standards).
The ICP-MS method validation covered the evaluation of limit of detection (LOD), the limit of quantification (LOQ), trueness, precision (repeatability). Moreover, the quality control sample at the concentration level of 50 $mg \cdot L^{-1}$ for Fe, and 500 $mg \cdot L^{-1}$ for Au was analysed every ten samples to ensure the quality of the routinely acquired results.
Linearities of calibration curves were evaluated within a range from 10 to 2 000 $\mu g \cdot L^{-1}$ for Fe and 100 to 10 000 $\mu g \cdot L^{-1}$ for Au, respectively. LODs and LOQs were calculated using the equations: LOD = 3.3 SD/s and LOQ = 10 SD/s, where SD is the standard deviation of the signal intensity (standard deviation of the intercept) and s is the slope of the calibration curve. Trueness and precision were assessed by analyses of 6 independently prepared spiked samples at the concentration level of 500 $\mu g \cdot L^{-1}$ for Fe, and 5 000 $\mu g \cdot L^{-1}$ for Au. Calculated recoveries for Fe, and Au in repeatedly measured QC samples (n=9) were 101.7 %, 101.8 %. The validation results are summarized in Table SI-5.

Table S5. Validation results for ICP-MS.

**Parameter**	**Analyte**	
	**Fe**	**Au**
**Calibration range [$\mu g \cdot L^{-1}$]**	10 – 2 000	100 – 10 000
**Correlation coefficient**	0.9999	0.9999
**LOD [$\mu g \cdot L^{-1}$]**	9	2
**LOQ [$\mu g \cdot L^{-1}$]**	27	6
**Trueness [%]**	96.3	101.1
**Precision [%]**	0.6	0.5

Table S6. Contents of Au and Fe in many independently prepared AuBSA-Fe samples as determined by ICP-MS and calculation of Au:Fe ratios in real samples.

Sample	[Au] [mM]	[Au] [mg·mL^{-1}]	[Fe] [μM]	[Fe] [μg·mL^{-1}]	Au:Fe		
**M-8**	3.9	0.8	321.1	17.9	12.2		
**M-7**	4.9	1.0	359.6	20.1	13.5		
**M-6**	5.9	1.2	421.5	23.5	14.0		
**M-5**	7.0	1.4	508.8	28.4	13.8		
**M-4**	8.3	1.6	636.4	35.5	13.1		
**M-3**	10.6	2.1	750.2	41.9	14.2		
**M-2**	11.7	2.3	894.4	49.9	13.0		
**M-1**	13.9	2.7	1049.2	58.6	13.2		
**M1**	10.9	2.2	807.0	45.1	13.6		
**M2**	14.0	2.8	1020.4	57.0	13.7		
**M3**	16.1	3.2	1193.3	66.6	13.5		
**M4**	17.1	3.4	1248.5	69.7	13.7		
				Average	13.5	13.3	Theory
				SD	0.5		

Note: Average Au:Fe ratio in real samples coincides well with the theoretical ratio of these metals (theoretical Au:Fe ratio derived from BSA:Au:Fe = 1:10:0.75 => 10/0.75 ≐ 13.3)

4. MRI for AuBSA-Fe samples

Table S7: Values of relaxation times T_1, T_2 and relaxation rates R_1, R_2 together with real iron concentrations (as determined by ICP-MS for concentrated samples, while derived from these values for diluted samples).

Sample	Sample concentration [%]	Fe concentration [mM]	T_1 [ms]	R_1 [s^{-1}]	T_2 [ms]	R_2 [s^{-1}]
M-8	100	0.321	2200	0.455	835	1.198
	75	0.241	2560	0.391	1085	0.922
	50	0.161	2285	0.438	809.5	1.235
	25	0.080	1490	0.671	492.2	2.032
M-7	100	0.360	2450	0.408	787	1.271
	75	0.270	2355	0.425	926.2	1.080
	50	0.180	3020	0.331	1360	0.735
	25	0.090	3390	0.295	1869.2	0.535
M-6	100	0.421	2305	0.434	700	1.429
	75	0.316	2570	0.389	926.2	1.080
	50	0.211	2800	0.357	1092	0.916
	25	0.105	3320	0.301	1765.3	0.566
M-5	100	0.509	1970	0.508	587.6	1.702
	75	0.382	2270	0.441	757.1	1.321
	50	0.254	2590	0.386	973.4	1.027
	25	0.127	3100	0.323	1579.4	0.633
M-4	100	0.636	1820	0.549	473	2.114
	75	0.477	2145	0.466	602.1	1.661
	50	0.318	2635	0.380	920.4	1.086
	25	0.159	3100	0.323	1420.8	0.704
M-3	100	0.750	1515	0.660	364	2.747
	75	0.563	1920	0.521	487.7	2.050
	50	0.375	2379	0.420	704.3	1.420
	25	0.188	2855	0.350	1151.2	0.869
M-2	100	0.894	1515	0.660	301.1	3.321
	75	0.671	1810	0.552	386.3	2.589
	50	0.447	2315	0.432	629.7	1.588
	25	0.224	2930	0.341	1003.1	0.997

Table S7. Cont.

**M-1**	100	1.049	1305	0.766	213.4	4.686
	75	0.787	1610	0.621	331.133	3.020
	50	0.525	2080	0.481	459	2.179
	25	0.262	2795	0.358	889.73	1.124
**M1**	100	0.807	1815	0.551	447	2.237
	75	0.605	2050	0.488	593	1.686
	50	0.404	2355	0.425	723	1.383
	25	0.202	2780	0.360	1023	0.978
**M2**	100	1.020	1655	0.604	379	2.639
	75	0.765	1810	0.552	427	2.342
	50	0.510	2230	0.448	638	1.567
	25	0.255	2430	0.412	918	1.089
**M3**	100	1.193	1365	0.733	264	3.788
	75	0.895	1560	0.641	308	3.247
	50	0.597	1830	0.546	479	2.088
	25	0.298	2590	0.386	911	1.098
**M4**	100	1.249	1271	0.787	216	4.630
	75	0.936	1535	0.651	290	3.448
	50	0.624	1915	0.522	442	2.262
	25	0.312	2655	0.377	767	1.304

Note: Green values were excluded.

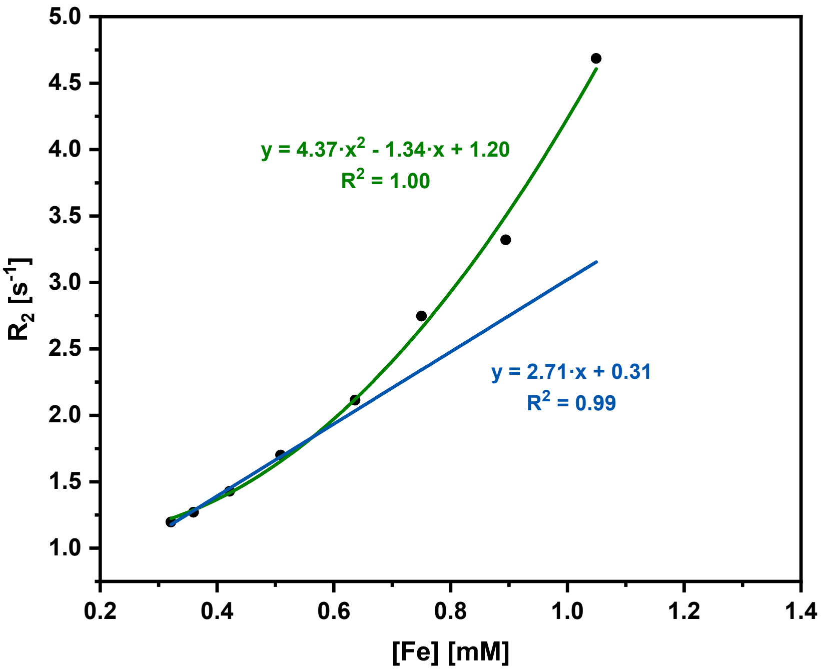

Figure S5. Relaxation rates as a function of iron concentration in AuBSA-Fe samples (100% concentration, any dilution is omitted). Comparison of linear and nonlinear (quadratic) fits.

Note: Quadratic fit is best suited for highly concentrated samples where the aggregation of protein occurs. Simultaneously, superparamagnetic iron oxide particles (SPIONs) attached to the protein are aggregated which may lead to the non-linear character of relaxation rate values with increasing sample concentrations.

5. Ageing and storage conditions of AuBSA-Fe samples

XPS spectra of AuBSA-Fe samples were measured shortly after their preparation (M6 in Figures SI-5) and after 1 year of ageing (M3 in Figures SI-5) at room temperature. Interestingly, in both samples only Au (0) was detected by XPS – see Figures SI-5**A** and SI-5**B**, which means that the inorganic part of the samples is not destroyed/changed. On the contrary, the relative contents of various types of organic species derived from N1s, O1s, C1s signals, varied significantly in fresh vs. aged samples – see Figures SI-5**C** – SI-5**J**. This points to a degradation of the organic part of AuBSA-Fe nanocomposites, namely a sort of oxidation takes place (based on occurrence of ammonium signal). It is highly recommended to store the samples in a fridge.

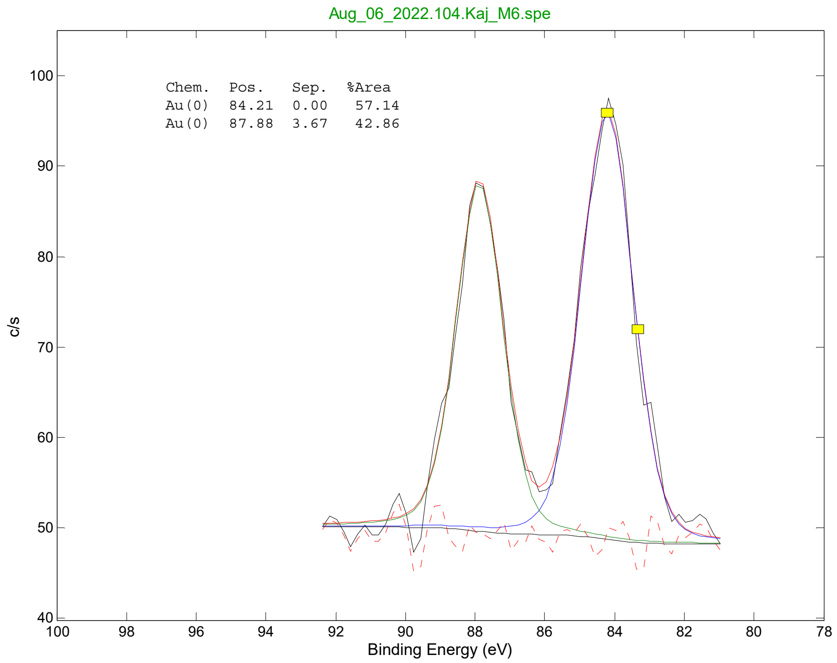

Figure S6A. XPS signal of fresh AuBSA-Fe sample, Au4f region.

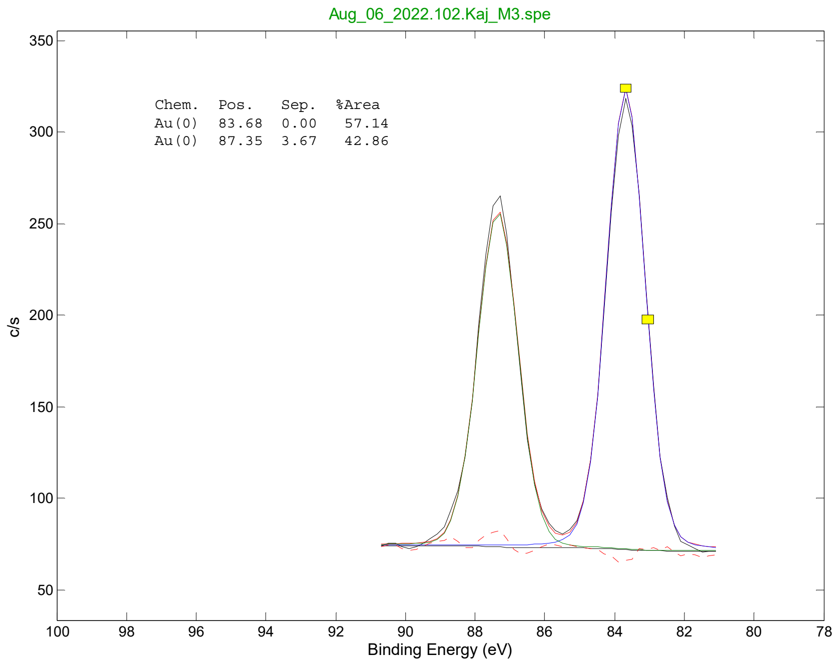

Figure S6B. XPS signal of one-year aged AuBSA-Fe sample, Au4f region.

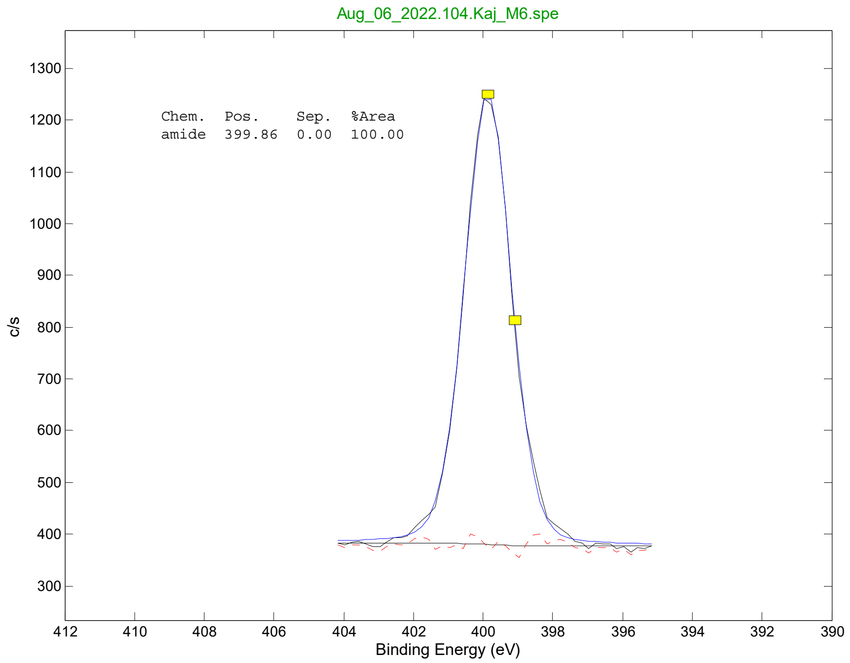

Figure S6C. XPS signal of fresh AuBSA-Fe sample, N1s region.

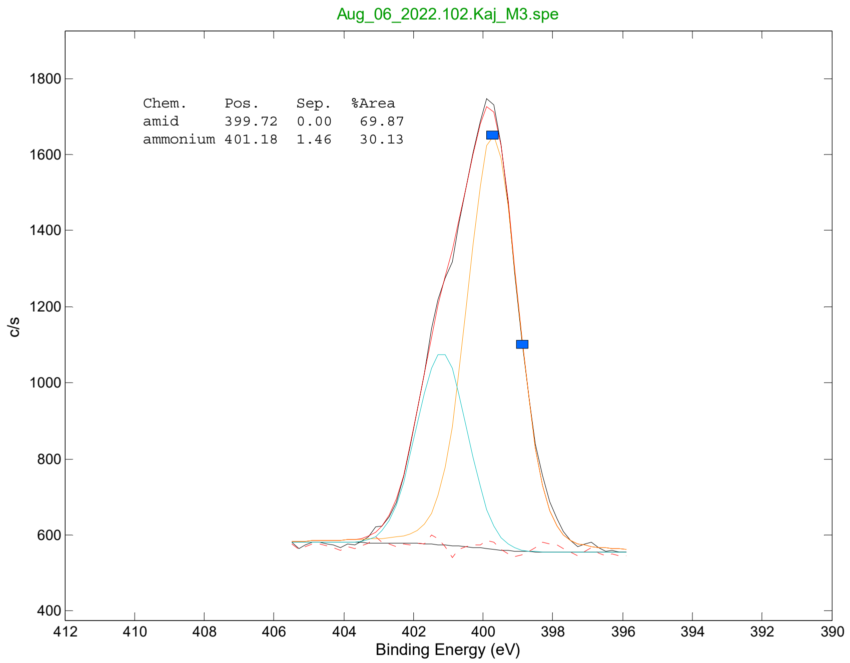

Figure S6D. XPS signal of one-year aged AuBSA-Fe sample, N1s region.

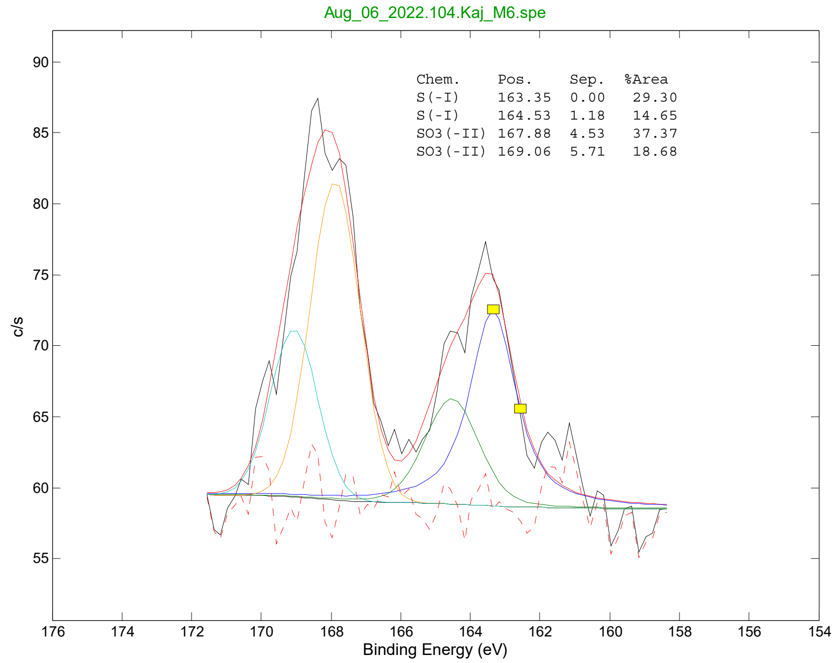

Figure S6E. XPS signal of fresh AuBSA-Fe sample, S2p region.

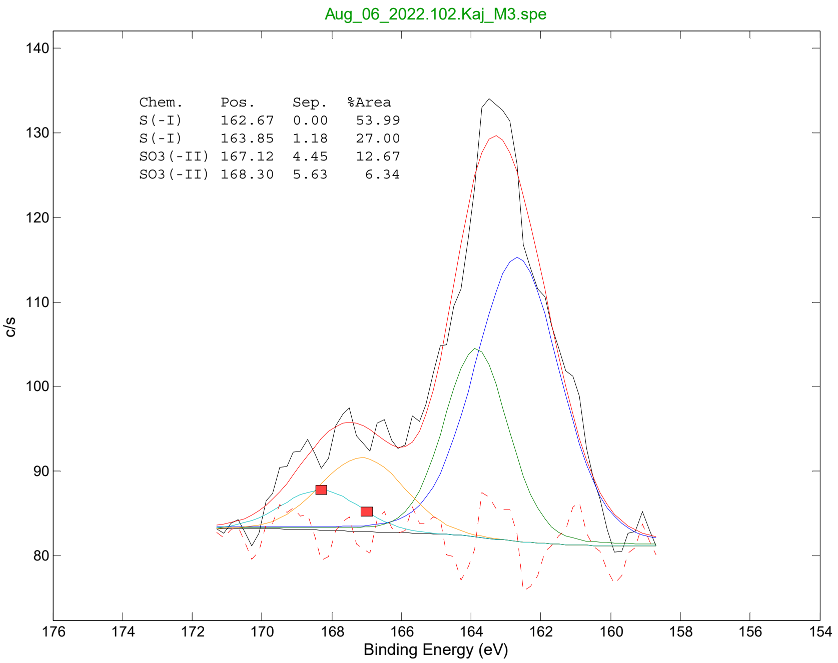

Figure S6F. XPS signal of one-year aged AuBSA-Fe sample, S2p region.

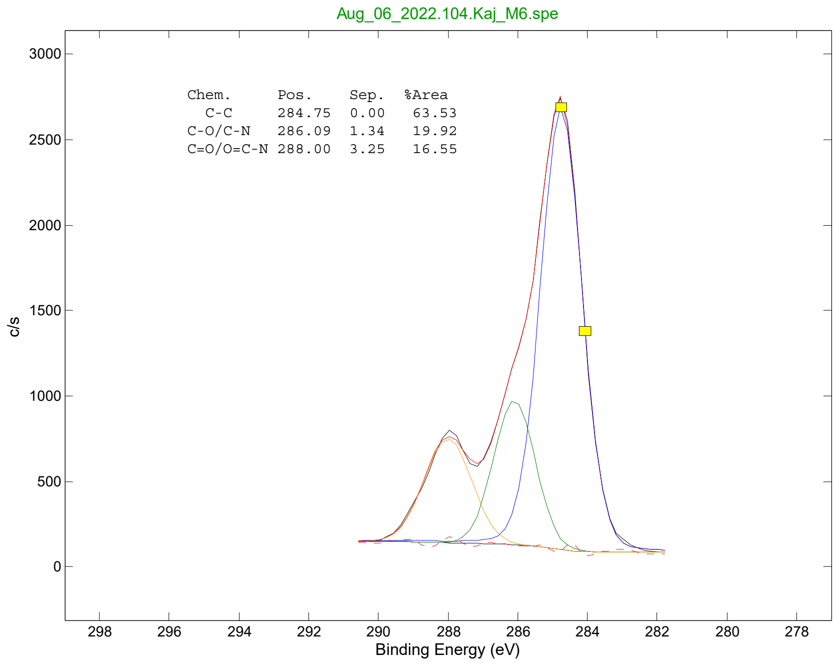

Figure S6G. XPS signal of fresh AuBSA-Fe sample, C1s region**.**

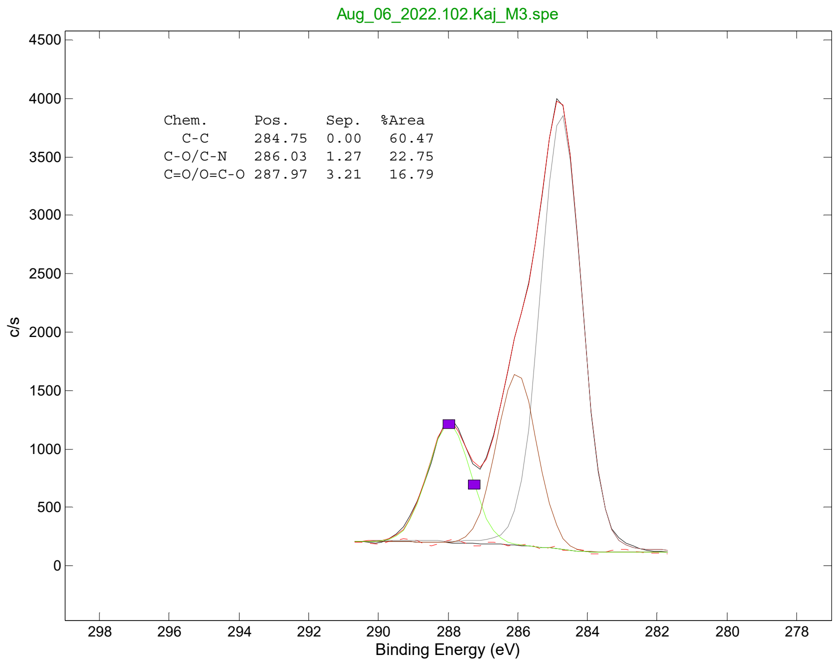

Figure S6H. XPS signal of one-year aged AuBSA-Fe sample, C1s region.

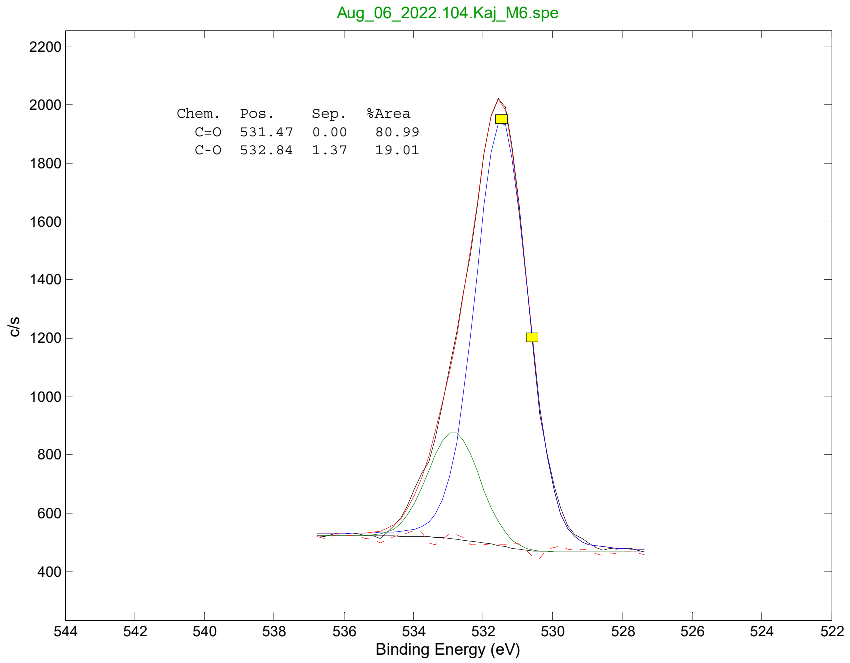

Figure S6I. XPS signal of fresh AuBSA-Fe sample, O1s region.

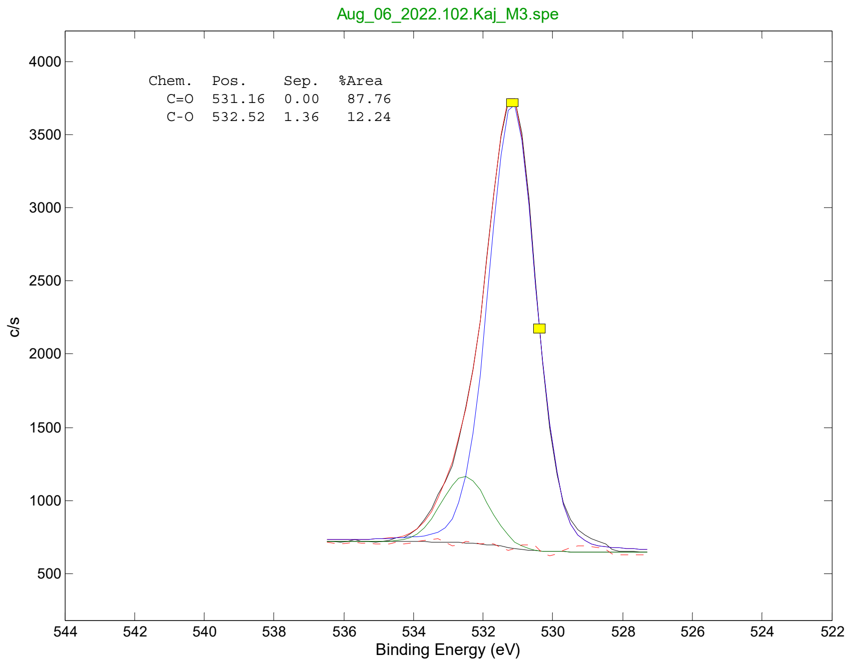

Figure S6J. XPS signal of one-year aged AuBSA-Fe sample, O1s region.

6. Cell viability tests of AuBSA-Fe nanocomposites – Alamar blue assay

Table S8: Table showing values of fluorescence in each well of the titration plate when cell viability tests of AuBSA-Fe nanocomposites and their precursors ($HAuCl_4$, mixture of $FeCl_2$ and $FeCl_3$) were performed in two representative iron concentrations (below and above 0.52 mM).

	1	2	3	4	5	6	7	8	9	10	11	12
A												
B		867	29735	2200	29289	24847	24735	1944	22831	24020	21247	
C		835	30876	2210	27409	23349	23919	1955	23864	24412	23791	
D		836	30373	2255	27282	23384	25086	1961	22664	24639	23549	
E		861	31141	2407	20874	23155	25072	2278	794	25358	24243	
F		856	30711	2354	20757	24421	24207	2288	801	25066	24370	
G		880	32978	2283	16906	24822	24941	2318	791	24956	24286	
H												

<table>
<tr><th></th><th>1</th><th>2</th><th>3</th><th>4</th><th>5</th><th>6</th><th>7</th><th>8</th><th>9</th><th>10</th><th>11</th><th>12</th></tr>
<tr><td>A</td><td></td><td></td><td></td><td></td><td></td><td></td><td></td><td></td><td></td><td></td><td></td><td></td></tr>
<tr><td>B</td><td></td><td rowspan="6">cultivation medium</td><td rowspan="6">RPE-1 cells</td><td rowspan="3">$HAuCl_4$</td><td rowspan="3">$FeCl_2$ + $FeCl_3$</td><td rowspan="6">AuBSA-Fe (c(Fe) < 0.52 mM)</td><td rowspan="6">AuBSA-Fe (c(Fe) ≥ 0.52 mM)</td><td rowspan="6" colspan="4">The other samples and precursors</td><td></td></tr>
<tr><td>C</td><td></td><td></td></tr>
<tr><td>D</td><td></td><td></td></tr>
<tr><td>E</td><td></td><td rowspan="3">$HAuCl_4$</td><td rowspan="3">$FeCl_2$ + $FeCl_3$</td><td></td></tr>
<tr><td>F</td><td></td><td></td></tr>
<tr><td>G</td><td></td><td></td></tr>
<tr><td>H</td><td></td><td></td><td></td><td></td><td></td><td></td><td></td><td></td><td></td><td></td><td></td><td></td></tr>
</table>

Note: For the detection of outliers (green values), Grubbs' or Dixon's test was used.
